# Highly efficient nonrigid motion‐corrected 3D whole‐heart coronary vessel wall imaging

**DOI:** 10.1002/mrm.26274

**Published:** 2016-05-25

**Authors:** Gastão Cruz, David Atkinson, Markus Henningsson, Rene M. Botnar, Claudia Prieto

**Affiliations:** ^1^ King's College London, Division of Imaging Sciences and Biomedical EngineeringLondonUnited Kingdom; ^2^ Centre for Medical ImagingUniversity College LondonLondonUnited Kingdom; ^3^ Pontificia Universidad Católica de Chile, Escuela de IngenieríaSantiagoChile

**Keywords:** nonrigid motion, coronary vessel wall, coronary MRA, image navigators

## Abstract

**Purpose:**

To develop a respiratory motion correction framework to accelerate free‐breathing three‐dimensional (3D) whole‐heart coronary lumen and coronary vessel wall MRI.

**Methods:**

We developed a 3D flow‐independent approach for vessel wall imaging based on the subtraction of data with and without T2‐preparation prepulses acquired interleaved with image navigators. The proposed method corrects both datasets to the same respiratory position using beat‐to‐beat translation and bin‐to‐bin nonrigid corrections, producing coregistered, motion‐corrected coronary lumen and coronary vessel wall images. The proposed method was studied in 10 healthy subjects and was compared with beat‐to‐beat translational correction (TC) and no motion correction for the left and right coronary arteries. Additionally, the coronary lumen images were compared with a 6‐mm diaphragmatic navigator gated and tracked scan.

**Results:**

No significant differences (*P* > 0.01) were found between the proposed method and the gated and tracked scan for coronary lumen, despite an average improvement in scan efficiency to 96% from 59%. Significant differences (*P* < 0.01) were found in right coronary artery vessel wall thickness, right coronary artery vessel wall sharpness, and vessel wall visual score between the proposed method and TC.

**Conclusion:**

The feasibility of a highly efficient motion correction framework for simultaneous whole‐heart coronary lumen and vessel wall has been demonstrated. Magn Reson Med 77:1894–1908, 2017. © 2016 International Society for Magnetic Resonance in Medicine

## INTRODUCTION

Coronary magnetic resonance angiography (MRA) has shown potential as a noninvasive diagnostic tool to assess the location and degree of lumen stenosis in coronary heart disease 1. Because coronary atherosclerosis is not necessarily stenotic 2 due to outward remodeling of the vessel wall 3, both lumen and wall assessments are desirable to more comprehensively detect coronary atherosclerosis. Atherosclerotic plaque burden increases as disease develops, often without significant changes to the vessel lumen. Because coronary plaque burden has been correlated with risk of future coronary events 4, direct and noninvasive visualization of the vessel wall is desired. A three‐dimensional (3D) flow‐independent approach for vessel wall imaging was proposed recently 5 based on an interleaved acquisition and subtraction of data with [T2prep(+)] and without [T2prep(−)] a T2‐preparation prepulse. This approach simultaneously provides coronary lumen [T2prep(+)] and vessel wall images; however, the required subtraction is particularly sensitive to respiratory motion corruption. Andia et al. 5 minimized respiratory motion using a one‐dimensional (1D) “pencil‐beam” diaphragmatic navigator gating 6, with data being accepted only when both T2prep(+) and T2prep(−) acquisitions were within the same small gating window of the respiratory cycle. This led to long and unpredictable acquisition times, because only a fraction of the acquired data was accepted for reconstruction (referred to as scan efficiency). Another limitation of 1D navigator gating is that only superior–inferior (SI) global translation can be corrected. Thus, motion from anterior–posterior (AP), right–left (RL), and nonrigid components remain uncorrected, which has been shown to be significant in some subjects 7, 8. Additionally, heart motion is estimated indirectly from the right hemi‐diaphragmatic displacement using a fixed linear correction factor of 0.6 9. It has been shown that the optimal factor varies for different regions of the heart and also for different subjects, meaning that motion artifacts may not be resolved fully if a fixed factor is used.

Several approaches have been proposed to address these limitations and compensate for motion in 3D coronary MRA. Subject‐specific scaling factors have been proposed to improve motion correction 10, 11 with no change in acquisition time. Model‐independent 1D self‐navigation techniques that repeatedly measure the k‐space center to infer the translational SI respiratory‐induced motion of the heart have also been proposed 12, 13. Because 1D self‐navigation methods measure a projection of the entire field of view, the estimated respiratory motion may be corrupted by signal contributions from static tissues such as the chest wall. Recently, image navigators (iNAVs) have been introduced to directly estimate the respiratory motion of the heart and allow separation of static tissues from the moving heart 14, 15, 16, 17, 18, 19, 20. Most of these approaches acquire a low‐resolution image navigator before the actual coronary MRA acquisition to correct two‐dimensional (2D) or 3D translational respiratory motion in a beat‐to‐beat fashion 15, 17. To account for more complex motion, “respiratory binning” techniques have been proposed 18, 21. Beat‐to‐beat approaches usually provide high‐temporal but low‐spatial resolution motion estimation, whereas binning approaches provide high‐spatial but low‐temporal motion estimation. In the binning approach, the acquired data are assigned to several states of the breathing cycle (or “bins”) and are later corrected to a reference position using the motion estimated from the binned images. 3D affine motion can be estimated from these bins and corrected in either k‐space 18 or directly in the reconstruction 21. Reconstruction‐based nonrigid correction has also been applied to cardiac MR using motion derived from training data 22 or coupled reconstruction problems 23. An alternative approach to nonrigid correction has also been introduced in the form of localized autofocusing techniques 24, 25, 26.

A recently introduced approach combined beat‐to‐beat 2D translational correction with bin‐to‐bin 2D affine correction for coronary lumen imaging 20. However, that approach does not correct for motion in the AP direction, and only affine correction is performed in the other two directions directly in the k‐space. In this study, we propose a combined motion correction approach for coronary lumen and vessel wall imaging using beat‐to‐beat (intra‐bin) 2D translational motion correction (RL and SI) and bin‐to‐bin (inter‐bin) 3D nonrigid motion correction. Motion‐corrected vessel wall images are obtained by acquiring a set of T2prep(+)/T2prep(−) data interleaved with a 2D iNAV in every heartbeat, which is used for translation correction and binning. Bins are reconstructed with soft‐gated 27 iterative SENSE 28 and nonrigid motion estimated via image registration 29 is incorporated into a motion‐compensated reconstruction 30. Subsequently, vessel wall images are obtained by subtraction as described by Andia et al. 5, producing a set of motion‐corrected, coregistered 3D coronary lumen and vessel wall images. The proposed method (2D translation + 3D nonrigid) was tested in 10 healthy subjects and compared with a diaphragmatic gated and tracked coronary MR angiogram, 2D translational motion correction, and no motion correction.

## METHODS

### Image Acquisition

**Figure 1:**
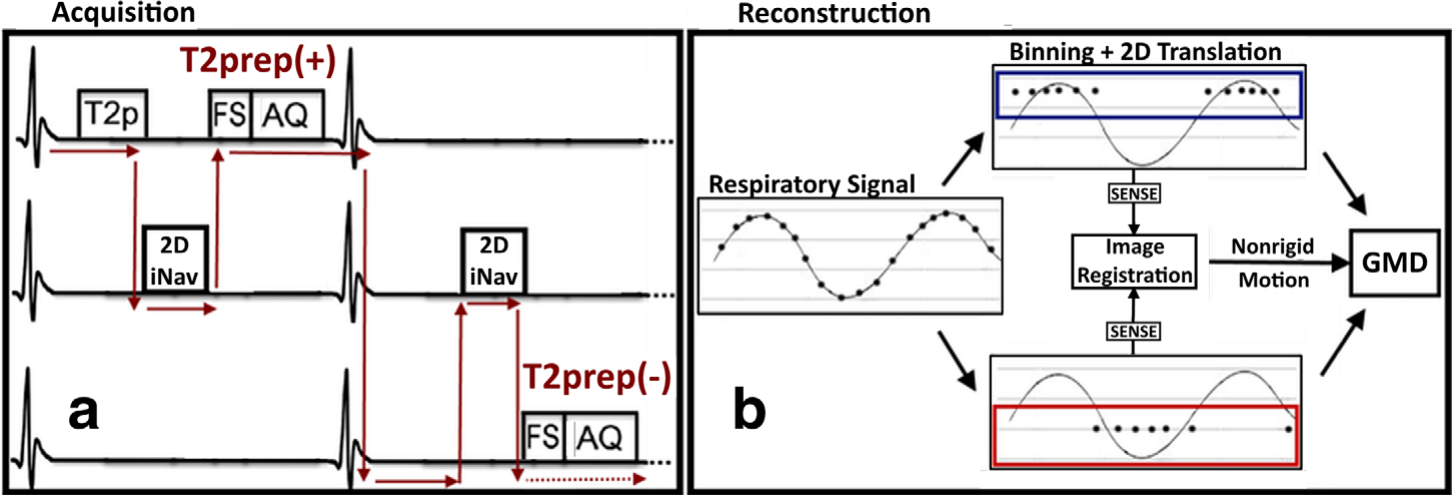
Framework of the proposed approach. (**a**) Acquisition: Data are acquired using interleaved scanning, allowing for datasets with and without T2 preparation [T2prep(+) and T2prep(−), respectively] to be acquired simultaneously with a 2D iNAV. In each heartbeat, a T2 preparation prepulse (T2p) is applied [in T2prep(+)], followed by the 2D iNAV, a spectral fat saturation pulse, and image acquisition (AQ). The arrows depict the way in which the three sequences interleave at runtime. (**b**) Reconstruction takes place in two steps. First, SI translation of the heart obtained from the 2D iNAVs is used to derive a 1D respiratory signal. Data are grouped into bins according to the respiratory position, and intra‐bin beat‐to‐beat translational motion is corrected in k‐space for each bin. Each binned dataset is reconstructed with soft gated iterative SENSE, and the resulting images are registered to retrieve a nonrigid motion field. Second, the motion fields are used in a GMD reconstruction to correct inter‐bin nonrigid motion.

Data were acquired using an interleaved scanning framework 31. Three scans were performed simultaneously: 3D segmented whole‐heart with T2 preparation [T2prep(+)], 3D segmented whole‐heart without T2 preparation [T2prep(−)], and 2D single‐shot golden radial coronal iNAV. The protocol was defined to acquire the T2prep(+) and T2prep(−) datasets at alternating heartbeats, with a 2D single‐shot golden radial iNAV being acquired every heartbeat (Fig. 1a). In every other heartbeat, a T2 preparation pulse was applied [for T2prep(+)], followed by the 2D iNAV and a fat saturation pulse, prior to 3D segmented data acquisition. The interleave scheme for the three scans is depicted in the sequence diagram of Figure 1a.

### Motion Estimation and Correction

The framework for motion estimation and correction was performed in two steps: 2D beat‐to‐beat translational motion correction followed by 3D bin‐to‐bin nonrigid motion correction, as shown in Figure 1b.

#### Beat‐to‐Beat Translational Motion Estimation and Correction

Beat‐to‐beat 2D translational motion was estimated from the iNAVs. Golden‐radial iNAVs undergo a gridding reconstruction 32 using an iterative density compensation function 33, providing a set of low spatial, high temporal–resolution images. A region of interest was selected around the heart 20 and rigid image registration was performed 34 to estimate SI and RL global translational motion. Outliers due to deep breaths were removed prior to any corrections. K‐space data with estimated SI motion ***T***_*SI*_ > μ + 2σ of the respiratory signal (where μ is the average respiratory position and σ is the corresponding standard deviation) were considered outliers and removed prior to reconstruction. Due to the difficulty of correcting and estimating motion in sparsely populated regions of the respiratory cycle, we opted to recover the approximately 5% most motion corrupted data points (outliers) via parallel imaging 28. The iNAV reference frame chosen for image registration corresponded to the average position at end‐expiration. SI motion information was also used to bin the 3D image data according to the position in the respiratory cycle. 2D global translational correction was applied within each bin separately by the corresponding phase shift in k‐space 35:

(1) $$K=\;K^{\prime }e^{2\pi ik^{\prime }\cdot T},$$

where ***K*** is the translation corrected k‐space, ***K*′** the acquired k‐space, ***k*′** the corresponding k‐space trajectory and ***T*** the estimated 2D translation vector.

#### Bin‐to‐Bin Nonrigid Motion Estimation

Bin‐to‐bin 3D nonrigid motion is estimated from the data itself. After translational correction, each 3D bin is reconstructed with a soft gating 27, 36 approach in which data are weighted according to their SI distance from the center of the bin, ***T*_SI_**. The binned, soft‐gated reconstruction can be formulated as

(2) $${\hat{I}}_{b}=\;arg\;min_{I_{b}}\{W_{b}{\left(EI_{b}-K_{b}\right)}_{2}^{2}\},$$

where ***I***_*b*_ represents the reconstructed bin volumes; ***W***_*b*_ is a diagonal matrix containing data weights for bin *b*; ***E*** is the encoding matrix that includes the Fourier transform, coil sensitivities and sampling operations; and ***K*** *b* represents the acquired data at each bin. The diagonal elements of ***W***_*b*_ were defined to be a linear function of the respiratory position of k‐space data as follows:

(3) $$w_{k}^{b}=\;\{\begin{array}{c} 1,\;if\;(R_{SI}(b)-T_{SI}^{b}(k)+r)/r>1\\ 0,\;if\;(R_{SI}(b)-T_{SI}^{b}(k)+r)/r<0\\ (R_{SI}(b)-T_{SI}^{b}(k)+r)/r,\;otherwise \end{array},$$

where $w_{k}^{b}$ represents the diagonal weights for data *k* at bin *b*, ***R***_*SI*_ (*b*) is the radius of bin *b* (i.e., the SI distance from the bin center to the edge), $T_{SI}^{b}$ (*k*) is the distance of k‐space point *k* to the center of bin *b*, and *r* is a parameter defining the range of the soft gate. Points within the bin have unity weight, decreasing linearly to zero as the distance of bin radius plus soft gate range is reached. Increasing *r* reduces undersampling artifacts at the expense of minor blurring. Bins were reconstructed with iterative SENSE 28. Image registration based on free‐form deformations 29 was performed, using the end‐expiration bin as reference 37, to estimate 3D nonrigid respiratory motion.

#### Translation Plus Nonrigid Motion Correction

Translational correction is applied directly in k‐space to correct intra‐bin motion, as described above (Equation (1)). Inter‐bin motion is corrected using the general matrix description (GMD) introduced by Batchelor et al. 30:

(4) $$\begin{array}{l} \hat{I}=arg\;min_{I}\left\{EI-K_{2}^{2}\right\}\\ E={\sum^{}}_{b}A_{b}FS_{c}U_{b}, \end{array}$$

where $\hat{I}$ is the motion‐corrected volume, ***K*** represents the translation corrected k‐space data, ***A***_*b*_ is the sampling matrix for bin *b*, ***F*** is the Fourier transform, ***S***_*c*_ represents the coil sensitivities for coil *c*, and ***U***_*b*_ represents the nonrigid motion fields obtained via image registration. The GMD reconstruction was performed with a linear conjugate gradient method 38 using the relative residual as regularization to prevent noise amplification.

#### Coronary Vessel Wall

Coronary vessel wall was obtained via image subtraction of the T2prep(+) from the T2prep(−) datasets, as described by Andia et al. 5, using

(5) $$I_{VW}=T2prep\left(-\right)-\;\lambda T2prep(+),$$

where ***I***_*VW*_ is the vessel wall image and *T2prep*(−) and *T2prep*(+) are the motion‐corrected images without and with T2 preparation pulse, respectively. The parameter *λ* is used to achieve maximum cancelation of signal from arterial blood. T2prep(−) and T2prep(+) were registered using nonrigid deformation 29 prior to image subtraction to guarantee spatial alignment. The optimum value for *λ* can be computed as a function of the heart rate and acquisition protocol as shown by Andia et al. 5.

### Experiments

#### Acquisition

Ten healthy subjects (age, 32 ± 8 y) were scanned under free‐breathing on a 1.5T clinical scanner (Philips Achieva, Philips Healthcare, Best, Netherlands) using a 32‐channel coil. Written informed consent was obtained from all subjects according to institutional guidelines, and the study was approved by our institutional review board. T2prep(+), T2prep(−), and iNAV data were acquired using an interleaved scanning framework 31. T2prep(+) and T2prep(−) data were acquired with an ECG‐triggered 3D balanced steady‐state free precession sequence using the following parameters: coronal slices; RL phase encoding; in‐plane resolution = 1 × 1 mm; slice thickness = 2 mm; field of view = 300 × 300 × 90 mm; repetition time/echo time = 5.3/2.6 ms; flip angle = 70 °; readout bandwidth (per pixel) = 433 Hz; subject‐specific acquisition window (range, 105.5–116.1 ms corresponding to 20–22 k‐space lines acquired per heartbeat); spectral fat saturation prepulse; subject‐specific mid‐diastolic trigger; and a low‐high (centric) Cartesian acquisition with radial‐like k‐space order in the k_y_ and k_z_ direction. The T2prep(+) acquisition included a T2 preparation pulse with a duration of 80 ms and two 180 ° adiabatic refocusing pulses. A 2D golden radial iNAV was acquired using a single‐shot spoiled gradient echo sequence using the following parameters: coronal slice (same geometry as the image data acquisition); in‐plane resolution = 4 × 4 mm; slice thickness = 25 mm; field of view = 300 × 300; repetition time/echo time = 2.4/1.07 ms; flip angle = 5 °; acquisition window = 47.2 ms with 24 angular profiles per cardiac cycle. Additionally, an ECG‐triggered 3D coronary MRA with diaphragmatic respiratory gating and tracking (6 mm gating window and tracking scaling factor of 0.6) was performed for comparison, using the T2prep(+) protocol as described above. The acquisition order of the proposed approach and the gated and tracked coronary MRA scan was randomized. The 3D coronary MRA protocol had a nominal scan time of approximately 10 min at 60 beats/min.

#### Reconstruction

Three reconstructions for coronary lumen and the vessel wall were obtained from the same acquired data: 1 non–motion‐corrected (NMC), 2 2D translational correction (TC), and 3 the proposed two‐step translational and nonrigid correction (TC+GMD). In addition, the 3D coronary MRA with a hemi‐diaphragmatic navigator and respiratory‐gated and corrected reconstruction (Gated) was used for comparison of coronary lumen images. The proposed method used the following (empirically chosen) parameters: three bins automatically defined such that each bin had the same amount of data; the soft gate range (*r*) was set to 1 mm to keep any residual motion smaller than the voxel size; the minimum relative residual of the iterative reconstructions was set to 0.05%. Four representative datasets (two male, two female) with SI respiratory amplitude (average and standard deviation) of 9.8 ± 2.4 mm were binned and reconstructed using three, four, and five bins. All reconstructions presented similar results upon visual inspection. Reconstructions were compared with the proposed method using three bins with metrics of lumen sharpness, vessel wall sharpness, and vessel wall thickness (described below). Data analysis was performed as described below; sharpness metrics were normalized to TC+GMD using three bins. One statistical difference (*P* < 0.01) was found between three bins and four bins. The remaining 17 quantitative metrics (Table 1) did not differ significantly, therefore three bins were used in this study to minimize computational time. The stopping criterion (relative residual) was determined by inspecting the noise amplification through different iterations of these bin reconstructions.

**Table 1:** Image Metric Results for TC+GMD Using Three, Four, and Five Bins

Image Metrics	TC+GMD		
	Three Bins	Four Bins	Five Bins
Lumen sharpness, a.u.			
LCA, full length	1.00 ± 0.26	0.96 ± 0.24	0.99 ± 0.25
RCA, full length	1.00 ± 0.14	0.99 ± 0.13	0.99 ± 0.16
LCA, first 4 cm	1.00 ± 0.17	0.96 ± 0.16	0.94 ± 0.19
RCA, first 4 cm	1.00 ± 0.28	0.99 ± 0.27	0.98 ± 0.28
LCA, mid	1.00 ± 0.47	0.96 ± 0.43	0.97 ± 0.44
RCA, mid	1.00 ± 0.19	0.96 ± 0.17	0.97 ± 0.21
Wall sharpness, a.u.			
LCA, full length	1.00 ± 0.09	1.09 ± 0.20	1.08 ± 0.27
RCA, full length	1.00 ± 0.37	1.07 ± 0.42	0.99 ± 0.33
LCA, first 4 cm	1.00 ± 0.16	1.10 ± 0.19	1.08 ± 0.24
RCA, first 4 cm	1.00 ± 0.40	1.08 ± 0.42^a^	0.99 ± 0.34
LCA, mid	1.00 ± 0.46	1.02 ± 0.54	1.01 ± 0.49
RCA, mid	1.00 ± 0.08	0.92 ± 0.15	0.92 ± 0.23
Wall thickness, mm			
LCA, full vessel	1.17 ± 0.15	1.21 ± 0.11	1.14 ± 0.12
RCA, full vessel	1.22 ± 0.16	1.17 ± 0.13	1.09 ± 0.09
LCA, first 4 cm	1.13 ± 0.15	1.18 ± 0.15	1.12 ± 0.13
RCA, first 4 cm	1.16 ± 0.15	1.16 ± 0.15	1.11 ± 0.11
LCA, mid	1.09 ± 0.16	1.13 ± 0.06	1.06 ± 0.11
RCA, mid	1.17 ± 0.18	1.07 ± 0.10	1.06 ± 0.06
Average reconstruction time, s	1680	2200	2720

a *P* = 0.01 versus TC+GMD/4 bins.

**P = 0.01 versus TC+GMD/5 bins.

Respiratory outliers were automatically removed before TC and TC+GMD reconstructions as described above. For the TC+GMD, each bin was translation‐corrected toward the central position of the bin. This translation‐corrected k‐space (i.e., the k‐space data of this group of bins) was reconstructed with the GMD 30 after nonrigid motion estimation. The TC approach used a 2D translational correction to a single reference followed by an iterative SENSE reconstruction, taking approximately 120 s. The proposed TC+GMD required a set of 2D translational corrected soft‐gated iterative SENSE bin reconstructions (∼490 s), followed by nonrigid image registration (∼170 s) and finally a motion compensated reconstruction (∼1020 s) for a total of approximately 1680 s. All reconstructions, image subtraction, and postprocessing were performed offline in MATLAB (MathWorks, Natick, Massachusetts, USA) on a PC with 12 CPUs (Intel Xeon 3.07 GHz). Coronary vessel wall images were obtained as described in Andia et al. 5, where a simulation study showed that the optimal λ for a balanced steady state free precession sequence lied between 1.21 and 1.27. Consequently, λ = 1.25 was used in this study for all subtractions. All reconstructions were reformatted onto a 2D plane using “Soap‐Bubble” software 39, facilitating the visualization of the right coronary artery (RCA) and left coronary artery (LCA). All image metrics were evaluated on reformatted images after 4 × zero‐padding (0.25 × 0.25 mm in‐plane reconstructed resolution).

#### Data Analysis

To evaluate the quality of motion correction, measures of vessel length, diameter, and sharpness were performed using Soap‐Bubble on the lumen of T2prep(+) images obtained with NMC, TC, TC+GMD, and gated reconstructions. Lumen vessel length and diameter were obtained by tracking the visible length of each coronary vessel. Lumen vessel sharpness was computed by taking the maximum gradient normalized to maximum center line intensity of profiles along the visible portion of the vessel. Lumen vessel sharpness was normalized to the mean sharpness of the reference gated acquisition. Lumen diameter and sharpness metrics were measured separately for the proximal section (the first 4 cm of the vessel), midsection, and full length.

To evaluate the impact of motion correction on the vessel wall images, metrics of vessel wall thickness and sharpness were computed on vessel wall images for NMC, TC, and TC+GMD. The vessel wall was not always visible in the distal sections of the coronaries; therefore, quantitative metrics for the full length were computed using predominantly the proximal and midsections. Vessel wall sharpness was obtained by taking the maximum gradient normalized to maximum intensity of profiles along the visible portion of the vessel wall, but tracking each side of the vessel wall of each coronary independently with Soap‐Bubble. Vessel wall sharpness was measured on a smaller vessel length than lumen sharpness because the wall was not always visible in the distal sections of the vessel, particularly on TC and NMC images. Vessel wall thickness was computed by first manually defining 20 1D profiles across the visible vessel wall of both RCA and LCA coronaries. Afterward, the average full width at half maximum of the selected profiles was computed to measure the vessel wall thickness. Vessel wall sharpness was normalized to the mean sharpness of the TC+GMD reconstruction. Vessel wall sharpness and thickness were measured separately for the proximal, mid, and full length coronary sections. Two experts (R.M.B. and M.H., with 20 and 7 years of experience in coronary MRA, respectively) blinded to the reconstruction methods were asked to score the sharpness of the coronary vessels on the following scale: 0, extreme blurring; 1, significant blurring; 2, some blurring; 3, minor blurring; and 4, no blurring. Visual assessment was performed on both T2prep(+) lumen and vessel wall images.

Statistical significance of the automated metrics (vessel sharpness, vessel diameter, vessel length, and vessel thickness) was evaluated with a paired *t* test (*P* < 0.01); statistical significance of the visual evaluations was performed with a Wilcoxon signed rank test (*P* < 0.01). This *P* value is stricter than that required by the Bonferroni correction on a *P* value of 0.05 for the coronary lumen and vessel wall comparisons (∼0.016 and 0.025, respectively). Statistical significance was tested against the Gated acquisition for lumen images and against TC+GMD reconstruction for vessel wall images.

## RESULTS

Scans were completed successfully in all subjects. Motion correction with insufficient quality to visualize the coronaries was obtained for one subject using the TC and TC+GMD approaches. This is thought to be due to the large SI respiratory amplitude of 30.8 mm and considerable cardiac motion in this subject. The gated acquisition had a scan efficiency of 29% in this case, and after two‐thirds of the scan all data were accepted due to low scan efficiency. This subject was treated as an outlier, and its results were not included in the statistics. The minimum, maximum, and average (and standard deviation) of the SI respiratory amplitude for the remaining subjects were 7.8, 17.8, and 11.0 ± 3.2 mm, respectively. The minimum, maximum, and average (and standard deviation) scan efficiencies for the gated acquisitions were 40%, 70%, and 59% ± 11%, respectively.

Metrics for lumen sharpness, vessel wall sharpness, and vessel wall thickness for the proposed TC+GMD using three, four, and five bins are shown in Table 1. Similar results were found with three, four, and five bins, except for a significant difference between the proximal vessel wall sharpness in the RCA between three and four bins. No significant differences were found for the remaining metrics; however, reconstruction time increased significantly when using additional bins. Three bins were used for all the remaining experiments in this study. For the proposed TC+GMD, the bin sizes (in millimeters) for the end‐expiration, mid‐cycle, and end‐inspiration bins were 2.49 ± 0.91, 2.30 ± 1.50, and 5.90 ± 2.24, respectively. The corresponding undersampling factors for these bins were 2.51 ± 0.42, 2.21 ± 0.35, and 3.15 ± 0.51, respectively.

**Figure 2:**
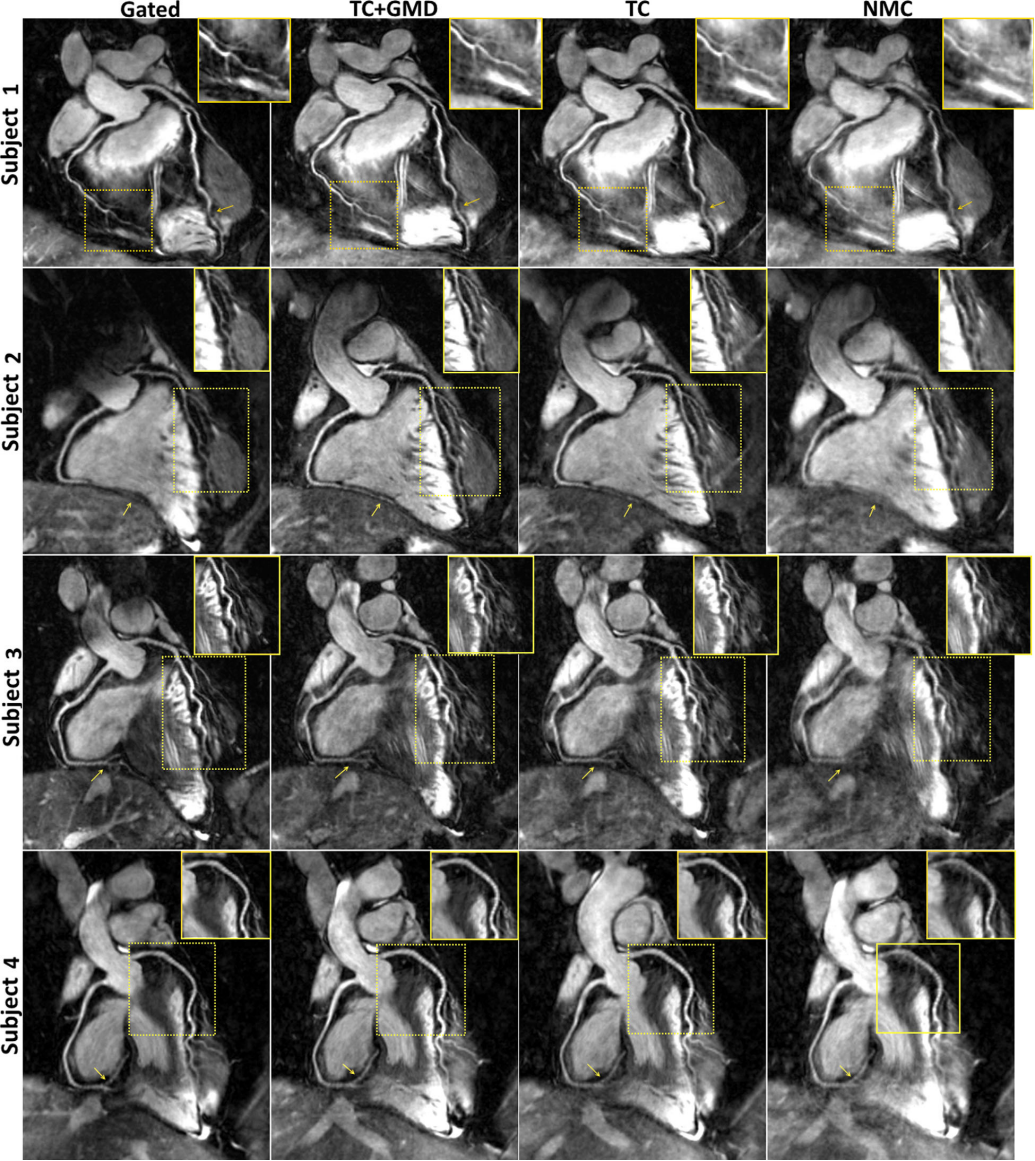
Reformatted coronary lumen images [T2prep(+)] for gated, TC+GMD, TC, and NMC for subjects 1–4. Blurring present in the NMC images is reduced with TC, and sharpness is further increased with TC+GMD (magnified boxes). The distal part of both coronaries is particularly affected by motion (arrows). Note that TC and TC+GMD have image quality similar to that for gated.

**Figure 3:**
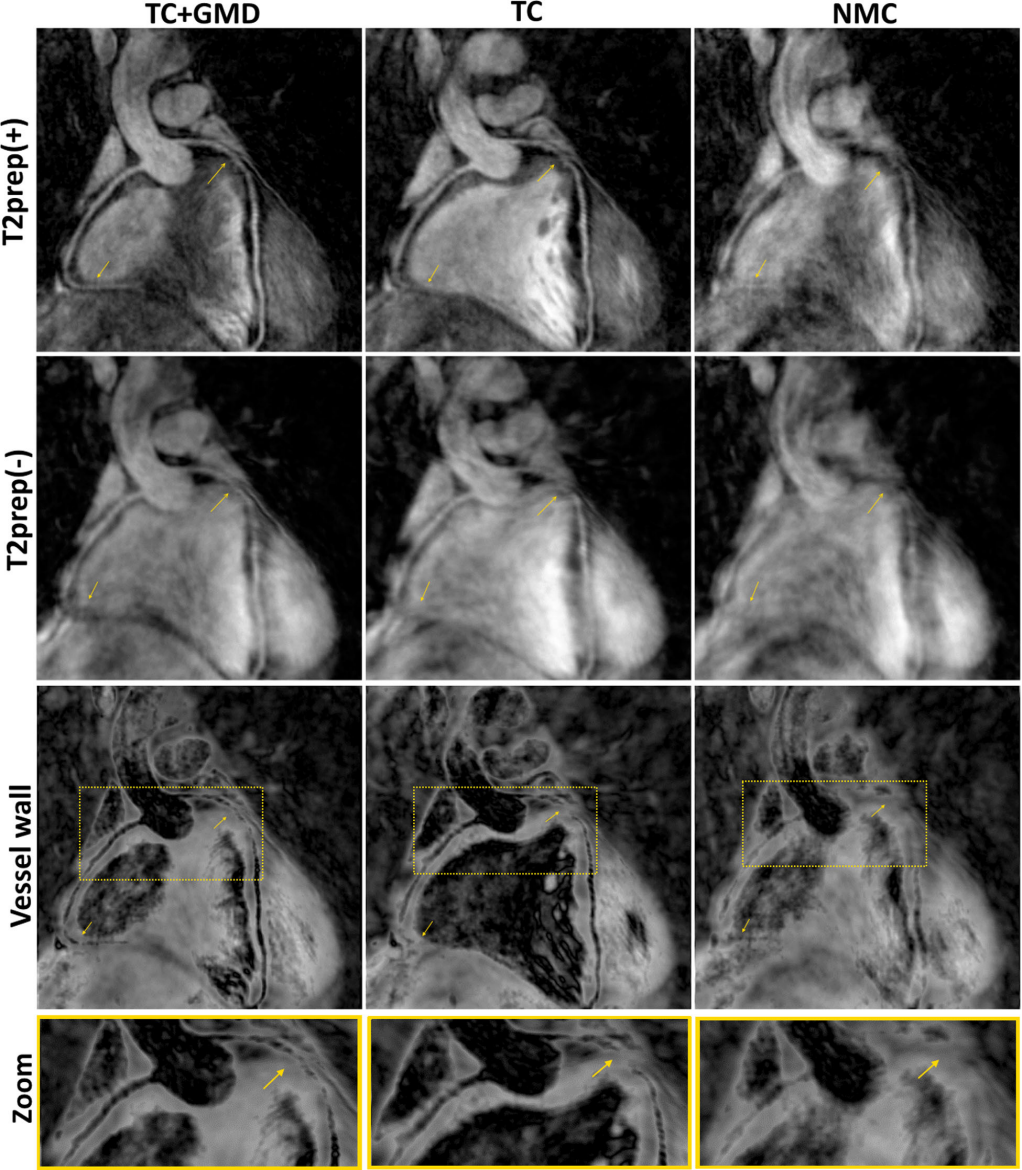
Reformatted vessel wall images for TC+GMD, TC, and NMC for subject 5. Significant motion artifacts can be seen in all NMC images. Most artifacts were removed with TC and further corrected with TC+GMD (arrows). Residual artifacts in either T2prep(+) or T2prep(−) contributed to blurring of the vessel wall and may have obscured it fully (magnified area in the Zoom row).

**Figure 4:**
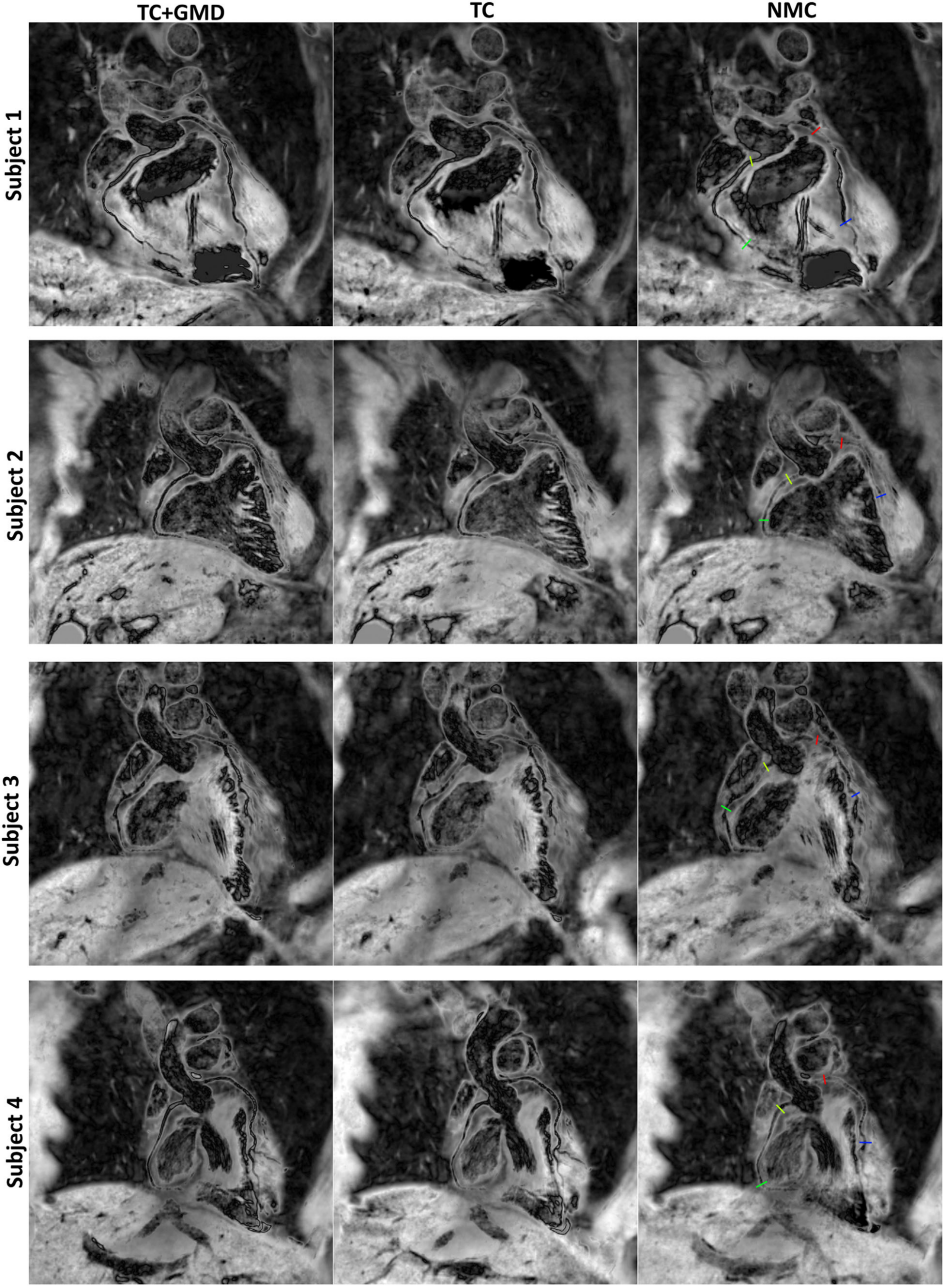
Reformatted vessel wall images for TC+GMD, TC, and NMC for subjects 1–4. The vessel wall is obscured in the NMC images. A significant improvement was obtained with TC, although small blurring remained. Vessel wall sharpness was further improved with TC+GMD. Colored lines in the NMC images mark the locations of the corresponding cross‐sectional views shown in Figure 5 for TC+GMD, TC, and NMC.

**Figure 5:**
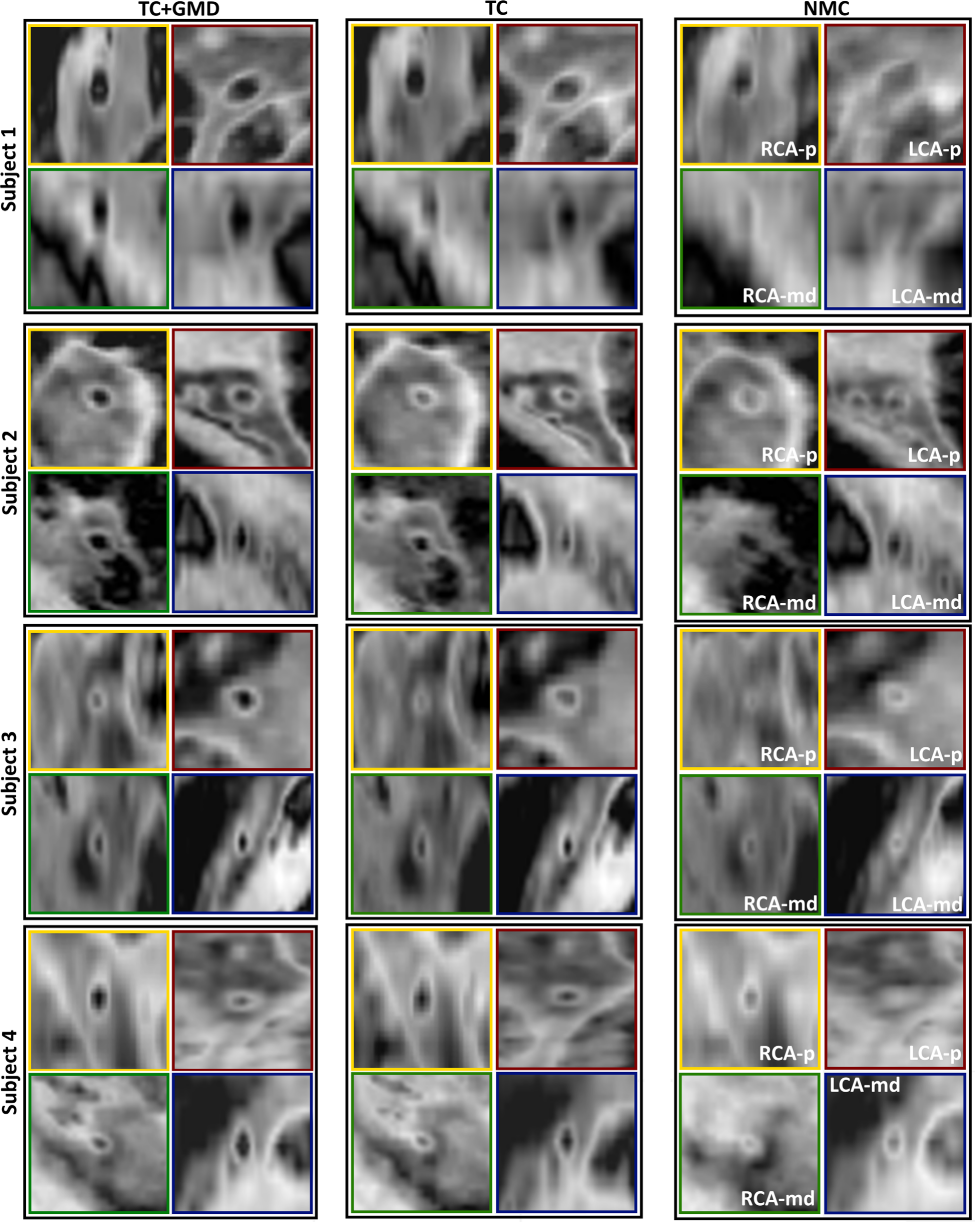
Cross‐sectional views of vessel wall images for TC+GMD, TC, and NMC for the corresponding subjects shown in Figure 4. For each subject, the RCA proximal (RCA‐p) is shown in the top left box (yellow), the RCA mid/distal (RCA‐md) is shown in the bottom left box (green), the LCA proximal (LCA‐p) is shown in the top right box (red), and the LCA mid/distal (LCA‐md) is shown in the bottom right box (blue). The vessel locations of the cross‐sections are shown in corresponding colors in Figure 4. The vessel wall is obscured in the majority of the cases; TC significantly improves visualization of the vessel wall and TC+GMD further improves the delineation of the vessel wall.

Reformatted images for ated, TC+GMD, TC, and NMC with T2prep(+) for subjects 1–4 are shown in Figure 2. Motion artifacts are visible in both coronaries in NMC, which are reduced by both TC and TC+GMD, although TC+GMD provides higher vessel sharpness in both the LCA and RCA (magnified box in subjects 1 and 2). These improvements are particularly visible in the distal part of the vessels (arrows in subjects 1 and 2). A set of T2prep(+)/T2prep(−) with the corresponding vessel wall image for subject 5 is shown in Figure 3. Most of the vessel wall in NMC is obscured by motion artifacts (arrows). Motion correction improves both T2prep(+) and T2prep(−) images, making the vessel wall visible in TC and better delineated with TC+GMD (magnified). Vessel wall images for TC+GMD, TC, and NMC for subjects 1–4 are shown in Figure 4. Corresponding cross‐sectional views for the locations in Figure 4 are shown in Figure 5. In Figure 4, the vessel wall for NMC appears blurred, whereas delineation of the vessel wall is considerably improved with TC and the sharpness of the wall further improved by TC+GMD. These improvements are clearly seen in the cross‐section views in Figure 5. The wall is not visible in most cases for NMC, becomes visible for most cases with TC, and appears better defined with TC+GMD. Residual artifacts are visible in the vessel wall images, originating from incomplete nulling of the blood pool in the subtraction. Inhomogeneities in the B0 and B1 fields cause the T2prep to be spatially dependent, leading to incomplete signal cancelation in some regions of the image.

**Figure 6:**
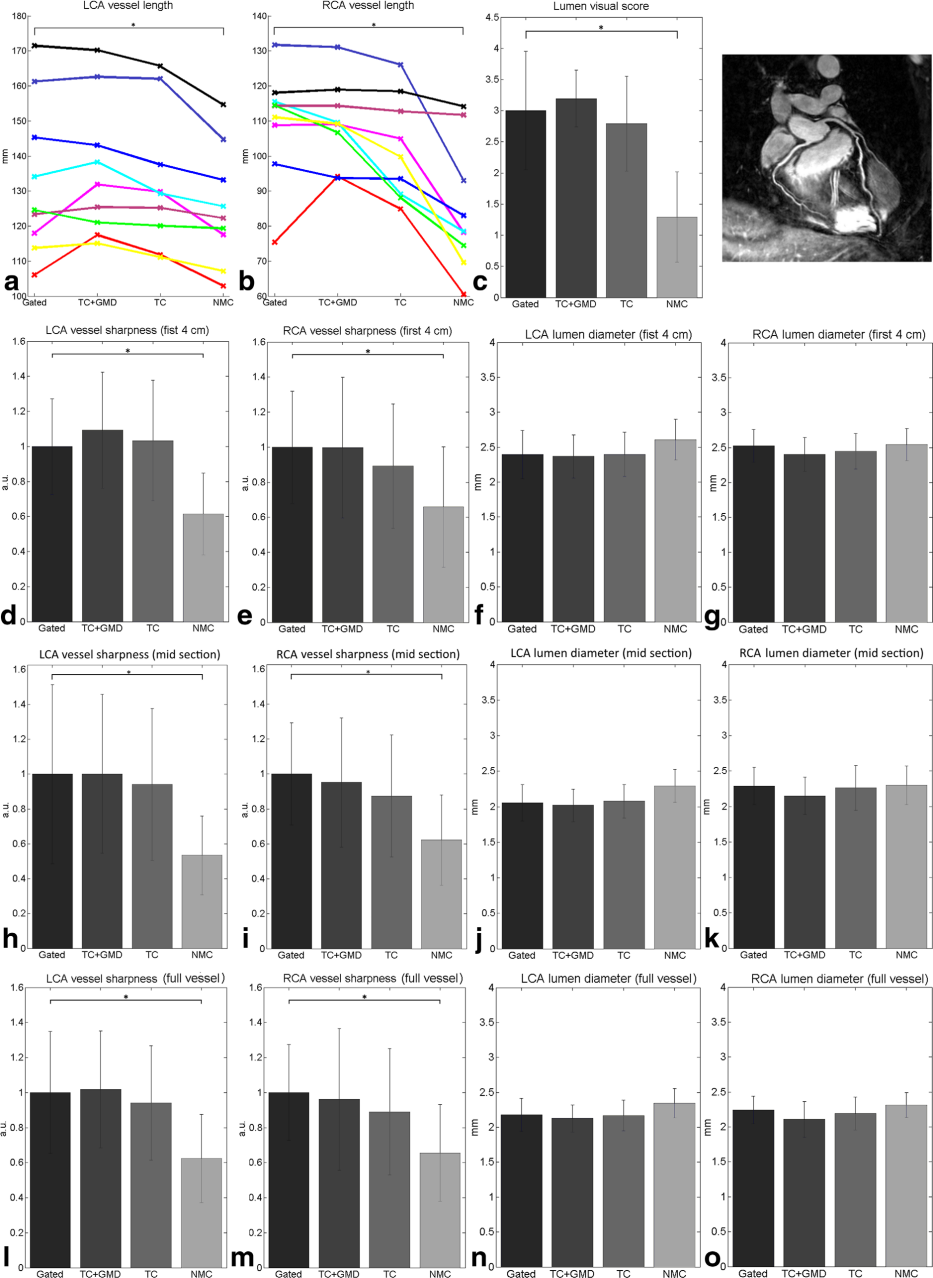
Image metrics for coronary lumen for nine subjects for gated, TC+GMD, TC, and NMC. (**a, b**) Vessel length along the LCA (a) and RCA (b) for nine subjects; each subject is assigned a different colored line. (**c**) Visual score of the coronary lumen images. (**d, h, l**) Vessel sharpness for the first 4 cm (d), midsection (h), and full length (l) of the LCA. (**e, i, m**) Vessel sharpness for the first 4 cm (e), midsection (i), and full length (m) of the RCA. (**f, j, n**) Lumen diameter for the first 4 cm (f), midsection (j), and full length (n) of the LCA. (**g, k, o**) Lumen diameter for the first 4 cm (g), midsection (k), and full length (o) of the RCA.*denotes a difference with *P* < 0.01.

Metrics for T2prep(+) coronary lumen evaluations are shown in Figure 6. Measured lumen vessel lengths were similar for gated and TC+GMD, with lower values for TC and NMC. Significant differences were found between gated and NMC for both coronaries. Lumen vessel length indicates reduced visibility of the distal part of both RCA and LCA when no motion compensation method is employed (NMC), showing gradual improvements with TC, TC+GMD, and gated. Similar results were found for lumen vessel sharpness, with significant differences between gated and NMC for both coronaries. TC+GMD had the smallest lumen diameter for both coronaries, whereas NMC had the largest due to blurring effects of respiratory motion. No significant differences were found in lumen diameter measurements. Lumen visual score results were as follows: 3.00 ± 0.95 for gated, 3.19 ± 0.46 for TC+GMD, 2.79 ± 0.76 for TC, and 1.29 ± 0.72 for NMC. NMC was significantly different from gated according to lumen visual score. The gated acquisition had an acquisition time of 1051 ± 66 s; NMC, TC, and TC+GMD had a fixed acquisition time of 620 s.

**Figure 7:**
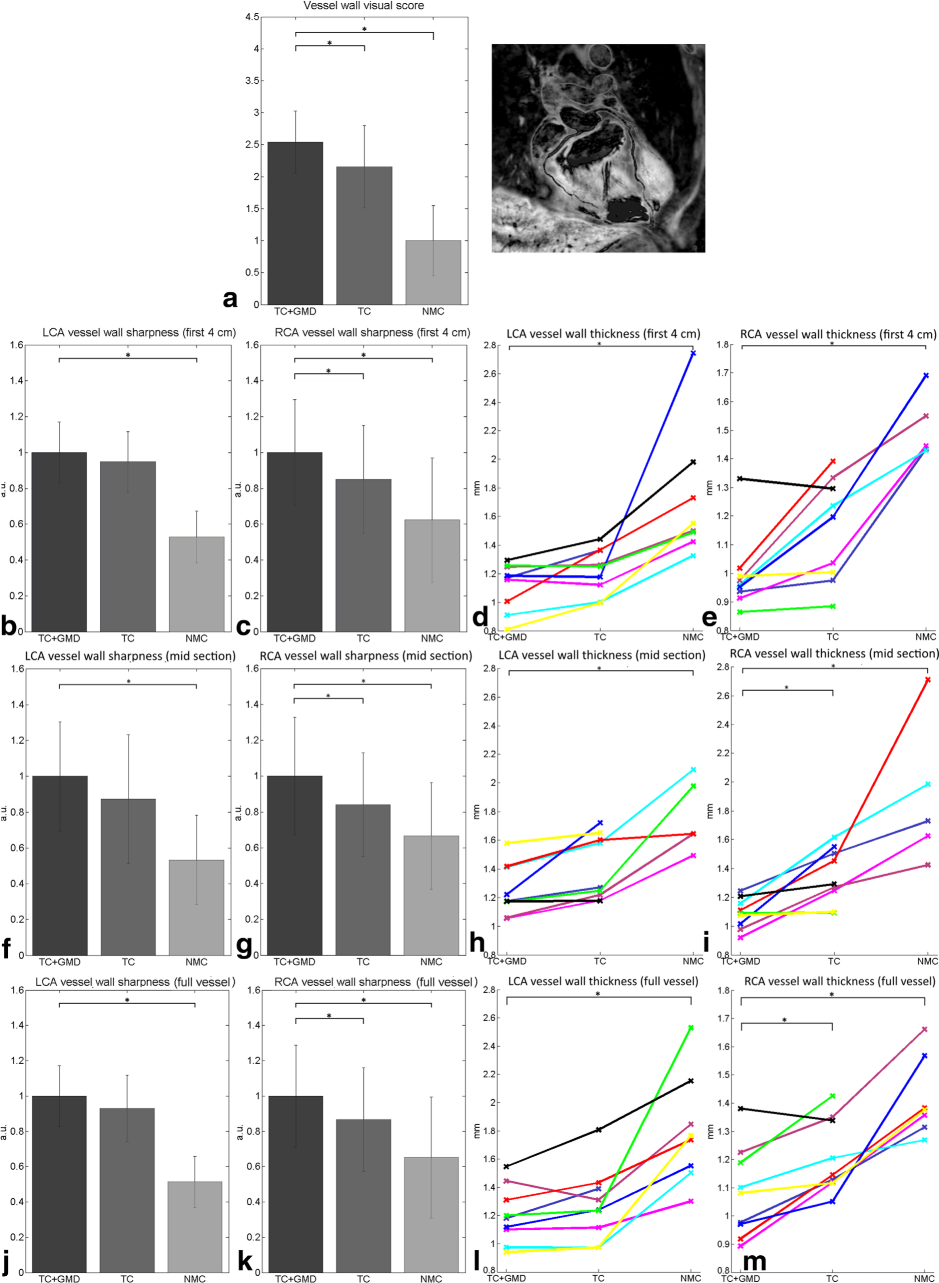
Image metrics for vessel wall images for nine subjects for TC+GMD, TC, and NMC. (**a**) Visual score of the vessel wall images. (**b, f, j**) Vessel wall sharpness for the first 4 cm (b), midsection (f), and full length (j) of the LCA. (**c, g, k**) Vessel wall sharpness for the first 4 cm (c), midsection (g), and full length (k) of the RCA. (**d, h, l**) Vessel wall thickness along the first 4 cm (d), midsection (h), and full length (l) of the LCA for nine subjects; each subject is assigned a different colored line. (**e, i, m**) Vessel wall thickness along the first 4 cm (e), midsection (i), and full length (m) of the RCA for nine subjects; each subject is assigned a different colored line.* denotes a difference with *P* < 0.01.

Metrics for coronary vessel wall analysis are shown in Figure 7. Blurring due to motion resulted in increased vessel wall thickness, as can be seen for NMC. Vessel wall thickness in NMC was not always visible. Out of the nine volunteers, NMC vessel wall thickness was not measured in two using the full length and was not measured in four using only the mid or proximal sections. The measured vessel wall thickness (in millimeters) using the full length for the LCA and RCA, respectively, were 1.20 ± 0.20 and 1.08 ± 0.16 for TC+GMD, 1.27 ± 0.26 and 1.21 ± 0.13 for TC, and 1.80 ± 0.39 and 1.42 ± 0.14 for NMC. Significant differences were found between TC+GMD and NMC for both coronary arteries and between TC+GMD and TC for the RCA using the full length and the midsection. Vessel wall sharpness measures were in agreement with lumen vessel sharpness metrics, however significant differences were found between TC+GMD and NMC for both coronaries and between TC+GMD and TC for the RCA. Similar results were found for the vessel wall visual score: 2.54 ± 0.49 for TC+GMD, 2.15 ± 0.64 for TC, and 1.00 ± 0.60 for NMC. Significant differences were found between TC+GMD and NMC and between TC+GMD and TC. Detailed results for all metrics are shown in Table 2. The corresponding *P* values for all metrics are provided in Supporting Table S1.

**Table 2:** Image Metric Results for Lumen and Vessel Wall Imaging

Image Metrics	Gated	TC+GMD	TC	NMC
Lumen vessel length, mm				
LCA	133.1 ± 22.1	136 ± 19.6	132.5 ± 19.7	125.3 ± 16.7^a^
RCA	109.7 ± 15.6	109.6 ± 11.6	101.9 ± 14.6	84.7 ± 18.3^a^
Lumen sharpness, a.u.				
LCA, full length	1.00 ± 0.35	1.02 ± 0.33	0.94 ± 0.33	0.63 ± 0.25^a^
RCA, full length	1.00 ± 0.27	0.96 ± 0.40	0.89 ± 0.36	0.65 ± 0.28^a^
LCA, first 4 cm	1.00 ± 0.27	1.09 ± 0.33	1.03 ± 0.34	0.61 ± 0.23^a^
RCA, first 4 cm	1.00 ± 0.32	0.99 ± 0.40	0.89 ± 0.35	0.65 ± 0.34^a^
LCA, mid	1.00 ± 0.51	1.00 ± 0.46	0.94 ± 0.44	0.53 ± 0.23^a^
RCA, mid	1.00 ± 0.30	0.95 ± 0.37	0.87 ± 0.35	0.62 ± 0.26^a^
Lumen diameter, mm				
LCA, full length	2.18 ± 0.24	2.13 ± 0.20	2.17 ± 0.22	2.36 ± 0.21
RCA, full length	2.24 ± 0.20	2.11 ± 0.25	2.19 ± 0.24	2.32 ± 0.18
LCA, first 4 cm	2.40 ± 0.35	2.37 ± 0.31	2.40 ± 0.31	2.61 ± 0.29
RCA, first 4 cm	2.52 ± 0.24	2.40 ± 0.25	2.45 ± 0.26	2.54 ± 0.23
LCA, mid	2.06 ± 0.25	2.02 ± 0.23	2.08 ± 0.23	2.29 ± 0.23
RCA, mid	2.29 ± 0.27	2.15 ± 0.27	2.26 ± 0.32	2.30 ± 0.27
Wall thickness, mm				
LCA, full vessel	NA	1.20 ± 0.20	1.27 ± 0.16	1.80 ± 0.39^b^
RCA, full vessel	NA	1.08 ± 0.16	1.21 ± 0.13^b^	1.42 ± 0.14^b^
LCA, first 4 cm	NA	1.12 ± 0.17	1.22 ± 0.16	1.72 ± 0.46^b^
RCA, first 4 cm	NA	1.01 ± 0.13	1.16 ± 0.18	1.51 ± 0.12^b^
LCA, mid	NA	1.25 ± 0.18	1.41 ± 0.23	1.77 ± 0.25^b^
RCA, mid	NA	1.09 ± 0.11	1.35 ± 0.19^b^	1.85 ± 0.46^b^
Wall sharpness, a.u.				
LCA, full length	NA	1.00 ± 0.17	0.93 ± 0.19	0.51 ± 0.15^b^
RCA, full length	NA	1.00 ± 0.29	0.87 ± 0.30^b^	0.65 ± 0.34^b^
LCA, first 4 cm	NA	1.00 ± 0.17	0.95 ± 0.17	0.53 ± 0.15^b^
RCA, first 4 cm	NA	1.00 ± 0.30	0.85 ± 0.30^b^	0.62 ± 0.35^b^
LCA, mid	NA	1.00 ± 0.30	0.87 ± 0.36	0.53 ± 0.25^b^
RCA, mid	NA	1.00 ± 0.33	0.84 ± 0.29^b^	0.67 ± 0.30^b^
Lumen visual score	3.00 ± 0.95	3.19 ± 0.46	2.79 ± 0.76	1.29 ± 0.72^a^
Vessel wall visual score	NA	2.54 ± 0.49	2.15 ± 0.64^b^	1.00 ± 0.60^b^
Scan efficiency, %	59 ± 11	96 ± 2	96 ± 2	100

NA, not available.

a *P* = 0.01 versus gated.

b *P* = 0.01 versus TC+GMD.

## DISCUSSION

A novel approach for nonrigid respiratory motion correction for 3D whole‐heart coronary MRI has been proposed and validated for simultaneous coronary lumen and vessel wall imaging. The proposed method uses a combined approach to motion correction: small amplitude motion is corrected with high temporal resolution intra‐bin translational corrections; large amplitude motion is corrected with low temporal resolution inter‐bin nonrigid motion correction. Translational motion is estimated from a golden radial 2D iNAV, whereas nonrigid motion is estimated from the data itself via binning and image registration. This approach was combined with an interleaved T2prep(+)/T2prep(−) acquisition, yielding motion corrected, coregistered, 3D coronary lumen and vessel wall images in a user‐defined fixed scan time.

Bin‐to‐bin 3D nonrigid motion correction was performed with the proposed TC+GMD approach after 2D (RL and SI) beat‐to‐beat intra‐bin translational correction. TC approach corrects only for 2D (RL and SI) beat‐to‐beat translational motion, thus AP motion remained uncorrected in the TC method. Both approaches were compared with a 6‐mm gated and tracked reconstruction. The TC+GMD approach in lumen imaging [T2prep(+)] showed improvements over TC and similar image quality to that for gated, while ensuring a predictable and highly efficient scan. No significant differences were found between gated and TC+GMD for lumen imaging. Whereas the proposed framework had a predictable scan time of approximately 1240 s (∼20 min) for a T2prep(+) and T2prep(−) dataset (i.e., lumen and vessel wall imaging), the corresponding gated approach 5 would be expected to require at least 2 × (1009 ± 68) s (∼35 min at 60 beats/min) plus additional time required for dual gating. For this reason, only a gated T2prep(+) data set was acquired in this study. The performance of the proposed TC+GMD on vessel wall imaging was compared with TC and NMC, showing improvements over both approaches. Significant differences were found between TC+GMD and TC for vessel wall imaging in terms of vessel wall visual score, RCA vessel wall thickness, and RCA vessel wall sharpness.

The proposed TC+GMD measured vessel wall thickness (full visible length) of 1.20 ± 0.20 mm and 1.08 ± 0.16 for the LCA and RCA, respectively. According to previous studies 40, 41, the coronary vessel wall is expected to be approximately 1.0–1.1 mm thick. The reason for the slight overestimation of wall thickness may be two‐fold: first, residual motion will manifest as blurring, increasing the apparent width of the vessels (and consequently the wall that is obtained by subtraction); second, the acquired spatial resolution (1 × 1 × 2 mm) may be insufficient to fully visualise the vessel wall and partial volume effects may occur, particularly in the distal part. Residual nonrigid motion may be addressed by increasing the number of bins in the framework. Here, the proposed TC+GMD was tested using three, four, and five bins, yielding similar results for different bins. Finer nonrigid motion correction is expected with increasing number of bins; however, undersampling artifacts also increase with the number of bins and may compromise motion estimation accuracy. For high number of bins, additional regularization in the bin reconstruction may be required (e.g., compressed sensing) to guarantee the reconstructions have sufficient quality for reliable motion estimation. The vessel wall was also partially obscured in some regions due to incomplete nulling of the blood pool. Field inhomogeneities may cause the T2 preparation to vary in space 42, therefore leading to an imperfect subtraction. This problem could be improved by optimizing the λ parameter for each subject. If estimates of the B0 43 and B1 44 fields are available, then that information could be incorporated into a spatially varying λ. Alternatively, this issue could be alleviated by employing additional 180 ° refocusing pulses during the T2 preparation or reducing the duration of the T2 preparation. The last solution will produce a more spatially uniform subtraction at the expense of contrast in the vessel wall image.

In one subject with large respiratory amplitude (∼31 mm, subject 10) and considerable cardiac motion, the proposed motion correction approach was not successful in removing all respiratory motion artifacts for both coronary lumen and vessel wall images. To tackle cases of large respiratory amplitudes, the proposed method could be combined with some level of gating while still maintaining high scan efficiencies.

A coronal iNAV was chosen over a sagittal iNAV for this experiment, but in general any geometry and protocol can be used for interleaved scanning 31. Coronal orientation has been shown to produce a superior lung–liver interface leading to more accurate SI motion estimation 45. Additionally, a coronal iNAV can use the geometry of the T2prep(+)/T2prep(−), simplifying the planning of the acquisition. The current framework uses a 2D iNAV, meaning that beat‐to‐beat translational correction is unavailable in one dimension (this was AP for the experiments performed). This limitation can be overcome by extending the proposed approach to acquire a low‐resolution 3D iNAV before or after image acquisition. A preliminary study in 46 indicates that reliable motion estimation may be obtained from a 3D iNAV with a spatial resolution of 5 × 10 × 10 mm, acquired in approximately 81 ms. Long‐duration iNAVs could compromise the efficacy of preparation pulses; however, compressed sensing may also be used to reduce the acquisition time of the 3D iNAV. Arrhythmia rejection was not implemented for the current version of the framework; therefore, residual cardiac motion due to respiratory sinus arrhythmia 47 may still be present. (Arrhythmia rejection will be implemented in future studies.) Compressed sensing could be used alternatively to reconstruct undersampled data due to cardiac motion in order to maintain the same scan time.

Future studies will explore more isotropic spatial resolution to measure the vessel wall with increased accuracy. The increased acquisition time will be addressed by combining accelerated reconstruction with motion correction to tolerate higher undersampling factors in both the binning and motion‐compensated coronary lumen and vessel wall reconstructions, as recently proposed for 3D abdominal imaging 48. Further study on optimal λ values is desired to produce more homogenous blood nulling in the vessel wall image. Future studies will validate the proposed method in patient scans and compare it with alternative motion correction approaches.

## CONCLUSION

In conclusion, a framework for nonrigid respiratory motion correction of simultaneous coronary lumen and vessel wall imaging has been introduced, using an interleaved scanning and a combination of 2D translational and 3D nonrigid motion correction. The proposed method allowed for 96% scan efficiency on average, reducing scan times by approximately 1.6 × on average relative to a gated acquisition while maintaining similar image quality. No significant differences were observed in coronary lumen images between the proposed approach and the reference gated scans. Significant improvements in the RCA vessel wall sharpness, RCA vessel wall thickness, and vessel wall visual score were observed when comparing the proposed approach with translational motion correction alone.

## Supporting information


**Table S1.** *P* values for the comparison of lumen metrics against the Gated and comparison of vessel wall metrics against the TC+GMD in Table 2.
