# Supplementary material for: Highly efficient nonrigid motion‐corrected 3D whole‐heart coronary vessel wall imaging
Source: Magn Reson Med. 2016 May 25;77(5):1894–908. doi: 10.1002/mrm.26274 (PMC5412916; doi:10.1002/mrm.26274)
Supplement: Supplementary file 1 — Table S1. P values for the comparison of lumen metrics against the Gated and comparison of vessel wall metrics against the TC+GMD in Table 2. [file MRM-77-1894-s001.docx]

Supporting Table S1. P-values for the comparison of lumen metrics against the Gated and comparison vessel wall metrics against the TC+GMD in Table 2.

| P-values | Gated | TC+GMD | TC | NMC |
| --- | --- | --- | --- | --- |
| LCA lumen vessel length (mm) | - | 0.1678 | 0.7819 | 0.0052 |
| RCA lumen vessel length (mm) | - | 0.9885 | 0.0889 | 0.0015 |
|  |  |  |  |  |
| LCA lumen sharpness (full length) (a.u.) | - | 0.8496 | 0.4862 | 0.0053 |
| RCA lumen sharpness (full length) (a.u.) | - | 0.6717 | 0.1629 | 0.0010 |
| LCA lumen sharpness (first 4 cm) (a.u.) | - | 0.2871 | 0.6656 | 2x10^-4^ |
| RCA lumen sharpness (first 4 cm) (a.u.) | - | 0.9751 | 0.1808 | 0.0043 |
| LCA lumen sharpness (mid) (a.u.) | - | 0.9895 | 0.5445 | 0.0063 |
| RCA lumen sharpness (mid) (a.u.) | - | 0.6333 | 0.1680 | 0.0016 |
|  |  |  |  |  |
| LCA lumen diameter (full length) (mm) | - | 0.4334 | 0.8593 | 0.0662 |
| RCA lumen diameter (full length) (mm) | - | 0.0664 | 0.4801 | 0.1952 |
| LCA lumen diameter (first 4 cm) (mm) | - | 0.7586 | 0.9957 | 0.1525 |
| RCA lumen diameter (first 4 cm) (mm) | - | 0.0494 | 0.3117 | 0.7670 |
| LCA lumen diameter (mid) (mm) | - | 0.7344 | 0.8384 | 0.0191 |
| RCA lumen diameter (mid) (mm) | - | 0.0140 | 0.6945 | 0.8242 |
|  |  |  |  |  |
| LCA wall thickness (full vessel) (mm) | N/A | - | 0.0995 | 0.0019 |
| RCA wall thickness (full vessel) (mm) | N/A | - | 0.0038 | 3x10^-4^ |
| LCA wall thickness (first 4 cm) (mm) | N/A | - | 0.0427 | 0.0058 |
| RCA wall thickness (first 4 cm) (mm) | N/A | - | 0.0177 | 3x10^-4^ |
| LCA wall thickness (mid) | N/A | - | 0.0119 | 0.0055 |
| RCA wall thickness (mid) | N/A | - | 0.0033 | 0.0084 |
|  |  |  |  |  |
| LCA wall sharpness (full length) (a.u.) | N/A | - | 0.0356 | 6x10^-6^ |
| RCA wall sharpness (full length) (a.u.) | N/A | - | 3x10^-4^ | 7x10^-5^ |
| LCA wall sharpness (first 4 cm) (a.u.) | N/A | - | 0.2457 | 1x10^-5^ |
| RCA wall sharpness (first 4 cm) (a.u.) | N/A | - | 0.0023 | 2x10^-4^ |
| LCA wall sharpness (mid) (a.u.) | N/A | - | 0.0252 | 2x10^-4^ |
| RCA wall sharpness (mid) (a.u.) | N/A | - | 0.0033 | 2x10^-4^ |
|  |  |  |  |  |
| Lumen visual score | - | 0.4598 | 0.5125 | 2x10^-4^ |
| Vessel wall visual score | N/A | - | 0.0037 | 2x10^-4^ |
| Scan efficiency (%) | N/A | N/A | N/A | N/A |
|  |  |  |  |  |
